# The phylogenetic position of Acoela as revealed by the complete mitochondrial genome of *Symsagittifera roscoffensis*

**DOI:** 10.1186/1471-2148-10-309

**Published:** 2010-10-13

**Authors:** Adina Mwinyi, Xavier Bailly, Sarah J Bourlat, Ulf Jondelius, D Timothy J Littlewood, Lars Podsiadlowski

**Affiliations:** 1 Department of Evolutionary Biology and Ecology, University of Bonn, An der Immenburg 1, 53121 Bonn, Germany; 2 UPMC-CNRS, FR2424, Station Biologique de Roscoff, Place Georges Teissier, 29680 Roscoff, France; 3 Department of Invertebrate Zoology, Swedish Museum of Natural History, Box 50007, 10405 Stockholm, Sweden; 4 Department of Zoology, Natural History Museum, Cromwell Road, London SW7 5BD, UK

## Abstract

**Background:**

Acoels are simply organized unsegmented worms, lacking hindgut and anus. Several publications over recent years challenge the long-held view that acoels are early offshoots of the flatworms. Instead a basal position as sister group to all other bilaterian animals was suggested, mainly based on molecular evidence. This led to the view that features of acoels might reflect those of the last common ancestor of Bilateria, and resulted in several evo-devo studies trying to interpret bilaterian evolution using acoels as a proxy model for the "Urbilateria".

**Results:**

We describe the first complete mitochondrial genome sequence of a member of the Acoela, *Symsagittifera roscoffensis*. Gene content and circular organization of the mitochondrial genome does not significantly differ from other bilaterian animals. However, gene order shows no similarity to any other mitochondrial genome within the Metazoa. Phylogenetic analyses of concatenated alignments of amino acid sequences from protein coding genes support a position of Acoela and Nemertodermatida as the sister group to all other Bilateria. Our data provided no support for a sister group relationship between Xenoturbellida and Acoela or Acoelomorpha. The phylogenetic position of *Xenoturbella bocki *as sister group to or part of the deuterostomes was also unstable.

**Conclusions:**

Our phylogenetic analysis supports the view that acoels and nemertodermatids are the earliest divergent extant lineage of Bilateria. As such they remain a valid source for seeking primitive characters present in the last common ancestor of Bilateria. Gene order of mitochondrial genomes seems to be very variable among Acoela and Nemertodermatida and the groundplan for the metazoan mitochondrial genome remains elusive. More data are needed to interpret mitochondrial genome evolution at the base of Bilateria.

## Background

Acoels are marine, soft-bodied, unsegmented worms without hindgut and anus - the mouth opens to a central digestive parenchyma, a gut lumen is absent. Acoels move with their multiciliated epidermis although many are 'surprisingly muscular' [1]. Most of the species are free-living, some are ectocommensals. Several species from the subtaxa Sagittiferidae and Convolutidae form obligate symbioses with green algae [2], making them functional photoautotroph organisms. In traditional systematics the Acoela were considered to be representatives of the Platyhelminthes, due to their 'flatworm-like' features such as the ciliated epidermis, the frontal organ, neoblasts, hermaphroditic reproduction, biflagellate sperm, and a lack of body cavities (acoelomate structure), hindgut and anus [3,4]. Based on the ultrastructural characteristics of cilia, and the hypothesized reduction of gut and protonephridia, Ehlers (1985) combined Acoela with Nemertodermatida to form the Acoelomorpha. In his system the Catenulida form the sister group to all other Platyhelminthes (Euplatyhelminthes), which comprise the sister groups Acoelomorpha and Rhabditophora. However, the monophyly of the Platyhelminthes was soon questioned because of the weakness of these morphological characters [5,6]. Subsequent ultrastructural studies have demonstrated numerous differences between Acoelomorpha and Platyhelminthes, particularly amongst characters once thought to be homologous. For example, frontal organ morphology [6,7], sperm ultrastructure [8], and patterns in the nervous and muscular systems [9-11] all demonstrate the uniqueness of acoelomorphs.

Early molecular systematic studies using ribosomal RNA genes strongly suggested that Acoela and Nemertodermatida were distinctly separate from the Platyhelminthes [12-14]; this result remains coherent even in the light of more taxa, more sequence data and more sophisticated models of phylogenetic analysis [15]. In the last 10 years several phylogenetic studies with molecular sequences have suggested a phylogenetic position of acoels as sister group to all other Bilateria [16-19]. Studies with broad taxon sampling of both Acoela and Nemertodermatida supported paraphyly of Acoelomorpha, with Acoela forming the sister group to the remaining Bilateria (Nemertodermatida + Nephrozoa) [19-21]. Presuming a position as sister group to all other Bilateria and considering the comparably simple body organisation, the morphological features of acoels may provide insights concerning the bodyplan of the 'last common bilaterian ancestor' [18], the ancestor of extant acoels, protostomes and deuterostomes [22]. Thus, acoels came into the focus of studies in evolutionary developmental biology as a possible window into the deep past of bilaterians [23-27].

In spite of these advances there is still controversy about the phylogenetic position of Acoela, and at the same time Nemertodermatida. Despite overwhelming molecular evidence against a platyhelminth affinity, some authors discuss the stem cell system of Acoela and Rhabditophora as a potential synapomorphy of these taxa [28]. However, data about stem cells from other invertebrate taxa are very sparse, so this character is in need of a broad comparative study. Recent phylogenomic studies do not recover platyhelminth affinities for Acoela, but show quite different results due to the varying amount of genes and taxa covered. A phylogenomic analysis of EST data from *Isodiametra pulchra *[29] found no relevant nodal support for any sister group relation. However, the best tree from this analysis clustered *I. pulchra *together with the deuterostomes. Another EST study, including the acoel species *Neochildia fusca *and *S. roscoffensis *also failed to support any convincing relationship with another metazoan phylum or lineage [30]. Thus, the authors omitted acoels from subsequent analyses due to their low leaf stability. Finally, a recent increase in taxon sampling incorporated in the latter study, with additional sampling of acoels and including nemertodermatids, supported Acoelomorpha (Acoela + Nemertodermatida) as a monophylum with bootstrap support of 70% and 90% in two datasets of different sizes [31]. Acoelomorpha were the sister group to *Xenoturbella *in that study, but with only moderate bootstrap support from one of the two analysed datasets. *Xenoturbella *and Acoelomorpha together formed the sister group to all other Bilateria (= Nephrozoa), once again with merely moderate nodal support.

To evaluate the phylogenetic position of acoels using an independent set of molecular data we present the first complete sequence of a mitochondrial genome of a member of the Acoela, *Symsagittifera roscoffensis *(Graff, 1891). We describe gene content and tRNA secondary structure, compare the mitochondrial gene order to other taxa and show the results of a phylogenetic analysis with sequence alignments from mitochondrial protein-coding genes.

## Results and discussion

### Organisation of the genome and genes

**Figure 1:**
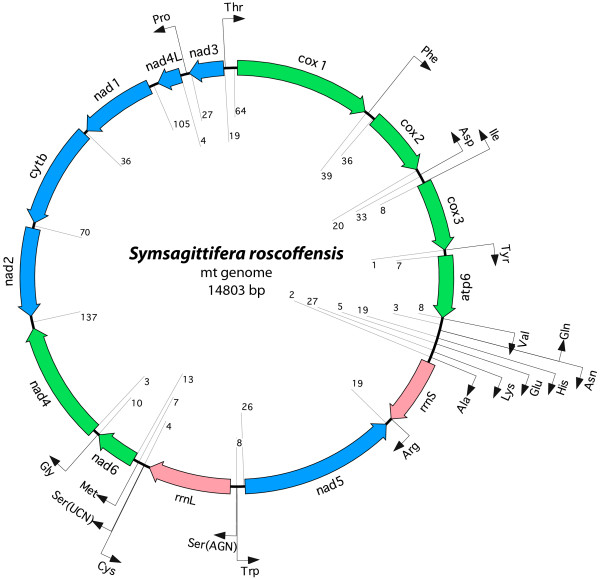
**Mitochondrial DNA map of *S. roscoffensis *[GenBank: **HM237350**]**. Gene abbreviations are explained in the text. Numbers show the size of non-coding regions between genes. Protein-coding genes on plus-strand are coloured in green, on minus-strand in blue and ribosomal RNA genes are in red. tRNA genes are black arrows, specified by the three letter abbreviation of the corresponding amino acid.

**Figure 2:**
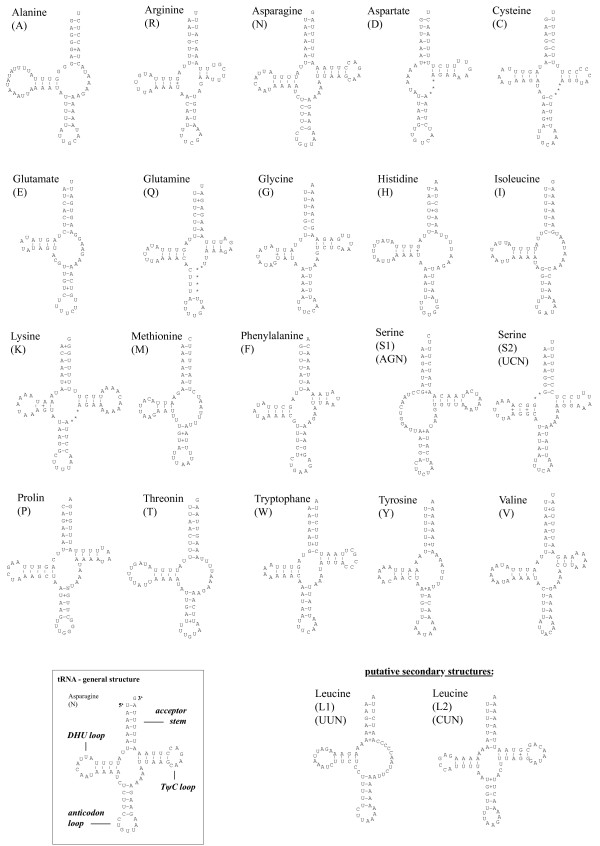
**Presumed secondary structures of the putative tRNA genes in *S. roscoffensis***. Sequences are illustrated from 5' to 3' end. *trnL1 *and *trnL2 *are nested in other genes (*nad5*, *rrnL*), therefore their existence is highly speculative. But there were no alternatives found in other parts of the mitochondrial genome.

The circular, double-stranded mitochondrial genome of *S. roscoffensis *consists of 14803 bp (Fig. 1, table 1). It contains two rRNA- and twelve protein-coding genes. The gene for *atp8*, which is normally also present in bilaterian mt genomes, is missing. This gene is also absent in the mitochondrial genomes of Platyhelminthes, Chaetognatha and almost all nematodes (except *Trichinella spiralis*). Thus there seems to be a tendency to lose *atp8 *in several unrelated taxa. We identified 20 tRNA genes and determined their putative secondary structures (Fig. 2). Despite a careful software search and inspection by eye, no sequence resembling the genes for *trnL1 *and *trnL2 *could be detected except within other genes. Two candidate positions for *trnL1 *and *trnL2 *are in *nad5 *(5778-5841; reverse direction) and in *rrnL *(8131-8201), respectively (Fig. 2). Amongst the tRNA genes, one loop of the typical cloverleaf structure is absent in some cases: *trnS1 *and *trnD *lack the DHU-stem. A missing DHU- stem in *trnS1 *is typical for all parasitic flatworms and many other Metazoa [32,33]. The TψC-stem is missing in *trnA*, -*H*, -*I*, -*M*, -*T*, -*E *and -*Y*. The majority of the tRNA genes show either mismatches of one to several nucleotides and/or shortened stems and enlarged loops, respectively (Fig. 2). Mitochondrial genes are transcribed from both strands, with *cox1-3*, *atp6*, *lrRNA*, *srRNA*, *nad6 *and *nad4*, as well as *trnT*, -*F*, -*Y*, -*V*, -*N*, -*H*, -*E*, -*K*, -*A*, -*R*, -*G*, -*S1, -S2 *and -*M *being transcribed from the plus-strand, the remaining ones from the minus-strand. Thirty, mostly short, non-coding regions can be found, ranging from one to 137 bp in length. None of these regions is significantly more AT-rich compared to the complete genome. Thus, a putative control region [34] could not easily be determined at first sight. The entire genome has a high A+T content of 75.3% and overall nucleotide frequencies of 38.6% A, 36.7% T, 12.8% G and 11.9% C. AT-skew [(A-T)/(A+T)]- and GC-skew [(G-C)/(G+C)] [35] of the whole plus-strand sequence are both close to zero (AT: 0.02; GC: 0.05), so there is no conspicuous strand-asymmetry in nucleotide frequency as in many other metazoan mt genomes [36]. High A+T content is a typical feature of nuclear ribosomal genes of Acoela, and it has been suggested a possible factor affecting phylogenetic resolution of these worms amongst the Metazoa [15], similarly to the acoels' highly truncated and modified rRNA genes.

**Table 1:** Mitochondrial genome organisation of *Symsagittifera roscoffensis*.

*Gene*	*Strand*	*Position* *(start - end)*	*Length (nuc.)*	*GC-/AT- skew*	*Start- codon*	*Stop- codon*	*Intergenic bp*
*cox1*	+	1 - 1551	1551	0.09/-0.16	GGT	TAA	39
*trnF*	+	1591 - 1651	61				36
*cox2*	+	1688 - 2428	741	0.08/-0.02	ATT	TAA	20
*trnD*	-	2449 - 2499	51				33
*trnI*	-	2533 - 2594	62				8
*cox3*	+	2603 - 3394	792	0.17/-0.2	ATT	TAG	1
*trnY*	+	3396 - 3451	56				7
*atp6*	+	3459 - 4160	702	0.04/-0.2	ATA	TAA	8
*trnV*	+	4169 - 4237	69				3
*trnQ*	-	4241 - 4303	63				0
*trnN*	+	4304 - 4375	72				19
*trnH*	+	4395 - 4456	62				5
*trnE*	+	4462 - 4527	66				27
*trnK*	+	4555 - 4621	67				2
*trnA*	+	4624 - 4688	65				0
*rrnS (12S)*	+	4689 - 5452	764	0.08/0.14			0
*trnR*	+	5453 - 5514	62				19
*nad5*	-	5534 - 7309	1776	0.05/-0.15	ATT	TAA	26
*trnW*	-	7336 - 7400	65				8
*trnS*-AGY	+	7409 - 7475	67				0
*rrnL (16S)*	+	7476 - 8417	942	0.27/0.1			0
*trnC*	-	8418 - 8478	61				4
*trnS-*UCN	+	8483 - 8546	64				7
*trnM*	+	8554 - 8602	49				13
*nad6*	+	8616 - 9095	480	0.01/-0.17	ATC	TAA	10
*trnG*	+	9106 - 9170	65				3
*nad4*	+	9174 - 10523	1350	0.12/-0.21	ATG	TAA	137
*nad2*	-	10661 - 11650	990	-0.07/-0.19	CAT	TAA	70
*cytb*	-	11721 - 12881	1161	0.03/-0.24	ATT	TAA	36
*nad1*	-	12918 - 13787	870	0.18/-0.25	ATG	TAA	105
*nad4L*	-	13893 - 14162	270	0.11/-0.07	ATG	TAA	4
*trnP*	-	14167 - 14234	68				27
*nad3*	-	14262 - 14654	393	0.14/-0.21	ATT	TAA	19
*trnT*	+	14674 - 14739	66				66

The whole molecule consists of 14803 nt; exact gene loci and the number of intergenic base pairs are depicted.

Overlaps between genes were not detected, except for the remaining possibility that *trnL1 *and *trnL2 *are positioned within *nad5 *and *rrnL*, respectively. All protein genes terminate with the codon TAA, except for *cox3 *ending with TAG. Existing start codons are more variable: ATT is found in *cytb*, *nad3*, *cox2 *and *cox3*; *nad1 *and *nad4 *start with ATG; *atp6 *begins with ATA and *nad6 *with ATC. Only *cox1 *with GGT and *nad2 *with CAT are exceptions from the commonly used start codons in mitochondrial genomes. Another uncommon feature is a repeat region of 42 bp found in *nad6 *(5' TGA GAA ATT TAC AAT CAA ATT TTA ACT ATT TCT CCT AGA TTT 3').

### Gene order

**Figure 3:**
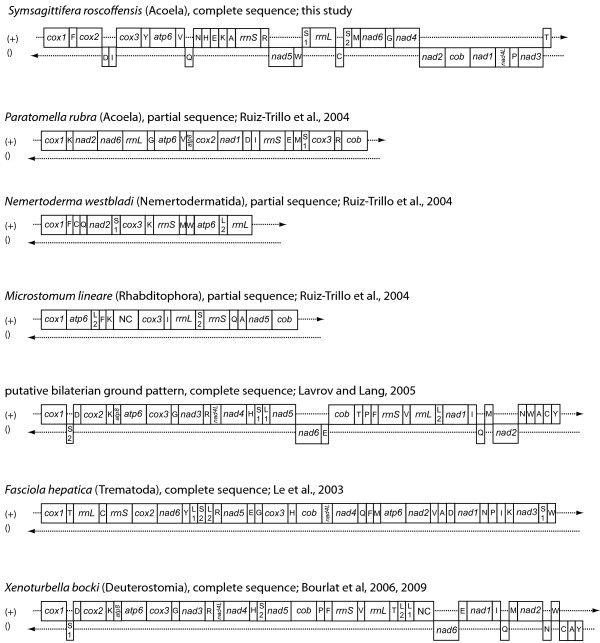
**Gene order comparison**. Gene order of *S. roscoffensis *compared to the partial sequences of *Paratomella rubra *(Acoela), *Nemertoderma westbladi *(Nemertodermatida) and *Microstomum lineare *(Rhabditophora), as well as to the complete sequence of *Fasciola hepatica *(Trematoda), *Xenoturbella bocki *(Xenoturbellida) and the putative bilaterian ground pattern.

The gene order found in *S. roscoffensis *shows no clear similarity with any other mitochondrial gene order published to date. Fig. 3 shows a comparison between *S. roscoffensis, Paratomella rubra*, *Nemertoderma westbladi *and *Microstomum lineare *[17], *Fasciola hepatica *[37], *Xenoturbella bocki *[38-40], and the putative bilaterian ground pattern [41]. Even the partial genome of *P. rubra *[17], another member of the Acoela, differs completely from our data. Both organisms share merely the fact that *rrnS *and *rrnL *are not adjacent, but separated by one (*S. roscoffensis*) and four (*P. rubra*) protein-encoding genes, respectively. This feature was also found in the nemertodermatid species *Nemertoderma westbladi *[17]. The ancestral state is supposed to be a separation only by *trnV *[41], a feature found in many metazoan mitochondrial genomes. Conserved gene blocks, which are shared with other taxa, could not be identified in *S. roscoffensis*. In addition to visual comparison of genome maps, we analyzed gene order data with CREx [42], determining the number of common intervals. This means the number of "blocks" with the same set of genes, regardless of their order inside a "block". We compared only gene orders of protein-coding and ribosomal RNA genes, as tRNAs are known to be subject to more frequent gene translocation than the larger genes. The result (Additional file 1, Fig. S1) shows overall low numbers of common intervals for comparison of *S. roscoffensis *with any of the other gene orders (0-18, whereas the maximum number of possible common intervals is 176). However, although not significant, the highest numbers (16-18) are obtained in comparison with the putative deuterostome ground pattern and with *Xenoturbella bocki *(which differ only by the relative position of *nad6*), while the lowest number was obtained in comparisons to platyhelminth gene orders (*Fasciola*: 2, *Schistosoma*: 0). We also determined breakpoint distances between these taxa, but these results were even less meaningful for *S. roscoffensis *(Additional file 1, Fig. S1).

### Phylogenetic analysis

**Figure 4:**
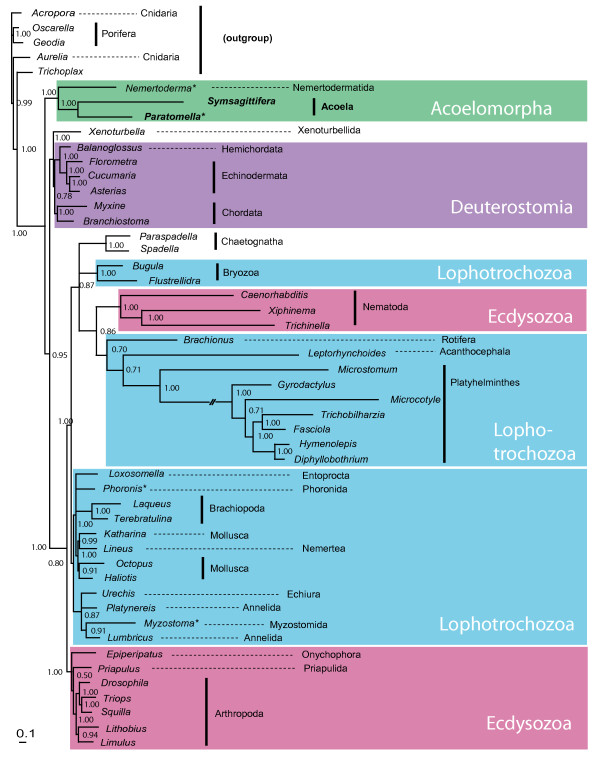
**Phylogenetic analysis of mitochondrial sequences - first alternative**. Best tree from two of four independent chains of Bayesian inference analysis (NH-PhyloBayes, CAT-BP, concatenated amino acid alignments of 11 mitochondrial protein-coding genes). Numbers close to nodes are Bayesian posterior probabilities. Branch lengths reflect substitutions per site (see scale bar). Asterisks indicate taxa with incomplete mt genome data.

Initial Maximum Likelihood (ML) and Bayesian Inference (BI) analyses with evolutionary models using site-homogenous substitution matrices derived from mitochondrial amino acid alignments (mtREV, mtZOA), yielded poor resolution of the phylogenetic position of acoels. It is well known that analyses of mitochondrial amino acid alignments on higher taxonomic levels suffer from two major problems: (a) the taxa may strongly vary in amino acid composition, which hampers the usefulness of a fixed substitution matrix, and (b) accelerated substitution rates in some fast-evolving taxa, leading to long-branch attraction artifacts. A few recent approaches aim to handle these problems more appropriately. In large datasets the empirical site-heterogeneous CAT mixture model [43] is superior to all site-homogenous amino acid matrix models in avoiding long-branch attraction [44]. In addition, changes in model parameters for parts of the tree will be applied empirically using the "breakpoint" (BP) approach in combination with the CAT model [45]. A recent study demonstrated the usefulness of the CAT-BP model by placing the long-branching Tunicata together with Acrania and Vertebrata as Chordata, while other model settings were not successful in this respect [46]. We set up a dataset of 50 species, including representative members of all phyla, among them long-branching taxa like Platyhelminthes and Nematoda. We ran four MCMC chains with NH-PhyloBayes and the CAT-BP option. Two chains converged with each other twice, each favoring different topologies with respect to acoels. In both variants Acoela and Nemertodermatida were supported as sister groups, with significant support values (Bayesian posterior probabilities equal or above 0.95). The first topology, recovered from two out of four independent chains (Fig. 4) shows significant support (1.0) for Acoela and Nemertodermatida forming the sister group to all other Bilateria (0.95). Ecdysozoa excluding nematodes (1.0) as well as Lophotrochozoa + Nematoda + Chaetognatha (1.0) and Deuterostomia + *Xenoturbella *(1.0) are significantly supported.

**Figure 5:**
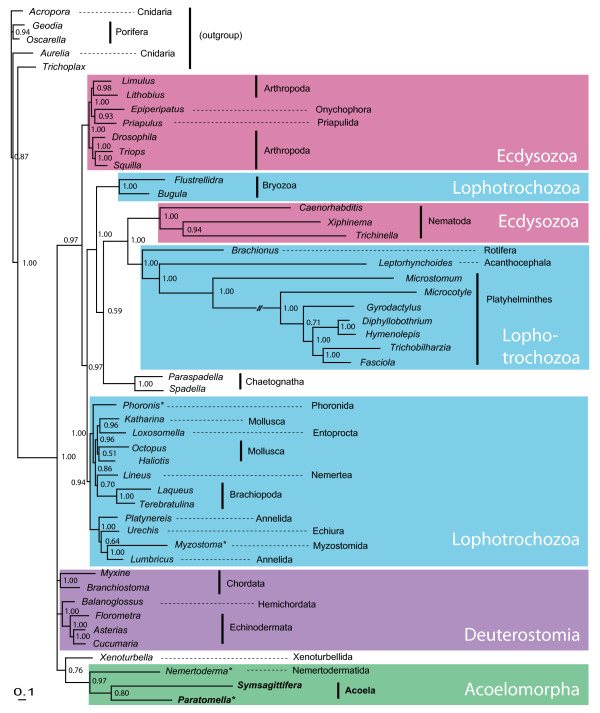
**Phylogenetic analysis of mitochondrial sequences - second alternative**. Alternative tree topology from one of four independent chains of Bayesian inference analysis (NH-PhyloBayes, CAT-BP, concatenated amino acid alignments of 11 mitochondrial protein-coding genes). Numbers close to nodes are Bayesian posterior probabilities. Branch lengths reflect substitutions per site (see scale bar). Asterisks indicate taxa with incomplete mt genome data.

The other topology, with small differences represented in the two other chains, found no resolution at the base of Bilateria (Fig. 5). Instead there is a polytomy of five taxa: *Xenoturbella*, Acoela + Nemertodermatida, Ambulacraria, Chordata and the protostomes. In the best tree *Xenoturbella *clusters with Acoela + Nemertodermatida, but there is no significant support for this group (0.76 and 0.92, respectively).

Due to these findings and the preliminary analyses we suspected that it was predominantly *Xenoturbella *which had an unstable position in the phylogenetic trees. Therefore we conducted additional analyses with a dataset without *Xenoturbella*, and a second analysis without Acoela and Nemertodermatida. In both variants four independent chains were run. In all four chains with *Xenoturbella *omitted, Acoela and Nemertodermatida form a monophylum which is the sister group to the remaining Bilateria (with significant support in three of the four chains; additional file 1, Fig. S2). In the analysis without Acoela and Nemertodermatida, *Xenoturbella *was found either as sister to Deuterostomia (with support values of 0.65 and 0.99) or as sister to Ambulacraria (with support values of 0.87 and 1.0)(Additional file 1, Fig. S3). Thus, in the absence of acoels *Xenoturbella *has a more unstable position in the bilaterian tree than acoels have in the absence of *Xenoturbella*. Acoels remain a critically important taxon to place within the Metazoa.

## Conclusions

### Concluding discussion

Mitochondrial gene order of the complete mitochondrial genome of the acoel *S. roscoffensis *is highly divergent from that of other bilaterian animals, including the partial mitochondrial genome of *Paratomella rubra*. Even computational approaches of gene order comparison like minimal breakpoint analysis and common interval analysis did not favour any affinity of *S. roscoffensis *to another taxon. Phylogenetic analyses of mitochondrial amino acid sequences give support for acoels forming a clade with nemertodermatids. But the limited available dataset representing the Nemertodermatida gives this result a rather preliminary nature. The dataset of *Nemertoderma westbladi *is incomplete as it consists of sequences from only three complete and two partial genes, thus covering only 34.8% of the final alignment. For a better evaluation of monophyly versus paraphyly of Acoelomorpha we are in need of more complete mitochondrial genome sequences from Nemertodermatida and Acoela.

Altogether we see more evidence for a position of Acoela and Nemertodermatida branching off early from the bilaterian tree rather than being grouped with deuterostomes or protostomes. The position of *Xenoturbella *cannot be fixed with this dataset, but its affinity to deuterostomes is greater than to Acoela and/or *Nemertoderma*.

Our trees also demonstrate limitations of the CAT-BP model with the bilaterian mitochondrial protein dataset. Nematoda are still clustering with the similarly long-branched Platyhelminthes and Syndermata, instead of forming a monophylum with other ecdysozoans (in this case arthropods, a priapulid and an onychophoran). Almost all other molecular datasets support Ecdyszoa (including Nematoda), so this must be an artifact probably due to selection. A similar problem is described from snakes, where selection seems to act on mitochondrial protein-complexes under special physiological conditions [47]. The long branches in the bilaterian tree are found in both parasitic and in free-living species of nematodes and platyhelminths. Thus, a parasitic life style does not seem to contribute to this accelerated evolutionary change.

If Acoela and Nemertodermatida represent ancient clades which split off early from the bilaterian tree, while *Xenoturbella *splits off later than these two, probably as sister group of the deuterostomes [38,40], then we can easily interpret morphological features shared by both as plesiomorphic character states, shared with the last common ancestor of Bilateria. As Telford [48] noted, Acoela and *Xenoturbella *share the following features: an acoelomate bodyplan with ventral mouth and absence of anus [1]; a unique tapering shelf at the ciliary tip and other similarities in the ciliary rootlets [49]; the nervous system is non-centralized and intra-epidermal in some Acoela and in *Xenoturbella *[50]. Recently, Nielsen [51] pointed out that the genomes of *Xenoturbella *and acoels have a significantly reduced Hox gene complement [25,26,52]. The more complex set of hox genes in the remaining bilaterians would be a valuable apomorphic character supporting Nephrozoa excl. *Xenoturbella*, a topology as in Fig. 5 (noting that there is only insufficient data from Nemertodermatida).

But this plesiomorphic feature cannot support a relationship between *Xenoturbella *and acoels. If *Xenoturbella *is part of the deuterostomes (as suggested by the tree in Fig. 4), the hox complement of *Xenoturbella *must be secondarily reduced, as there are many similarities in the hox complement of the remaining deuterostome and protostome taxa. Nuclear genome data of the complete hox clusters seem to be indispensible for a comprehensive evaluation of the evolution of hox genes at the base of Bilateria.

With regard to comparative analysis of mitochondrial genomes, especially gene order, more data are definitely needed, e.g. complete mitochondrial genome sequences from more than one acoel species, since the comparison of *S. roscoffensis *and *P. rubra *has shown that gene order in acoels seem to differ radically. Thus, single "representative" species are by no means sufficient to characterise or represent taxa [53]. Similarly, nemertodermatid genomes also require further and exhaustive evaluations. There is still no complete mitochondrial sequence of this group available, preventing meaningful, genome-based phylogenetic analyses.

## Methods

### Specimen, DNA extraction, PCR and cloning

*S. roscoffensis *specimens were provided by Xavier Bailly and were sampled close to the Station Biologique de Roscoff (France). All analyses were conducted with individuals preserved in 100% ethanol. DNA was isolated from whole animals (approx. 15-20 specimens per approach) using the DNeasy Blood & Tissue 50 kit (Qiagen, Germany) and following the manufacturer's guidelines. Based on previously published primers for *cox1 *[54], *cytb *[55] and *rrnL *[56] initial genome fragments were amplified and provided the basis for specific primer design. Additional primary sequence information came from the EST sets provided by the NCBI nucleotide database and NCBI trace archive. Primer suitability for both amplification and sequencing was checked with the NetPrimer tool (Premier Biosoft int.). Primer sequences and annealing temperatures are given in table 2. General PCR setup was done in 50 μl volumes (41.75 μl water, 5 μl 10× buffer, 0.25 μl Taq polymerase (5 U/μl), 1 ml dNTP mixture, 1 μl template DNA, 1 μl primer mixture (10 μM each)). PCR conditions were: 94°C for 2 min, 40 cycles of 94°C for 30 sec, primer-specific annealing temperature for 1 min, 68°C for 1 min and a final extension step of 68°C for 2 min. For standard PCR up to 3 kb we used the Eppendorf 5-prime Taq polymerase and buffer (5-prime, Germany). When we expected large PCR fragments we used the Takara LA kit and set up PCRs in 25 μl volumes (16.75 μl water, 2.5 μl buffer, 0.25 μl Takara LA Taq polymerase, 4 μl dNTP mixture, 1 μl template DNA, 0.5 μl primer mixture (10 μM each)). In this case conditions were as above except for the extension step of 72°C for 10 min at the end of each cyclic PCR run. The amplified products were checked on 1% TBE agarose gels and either purified directly with the Nucleo Spin Extract II kit (Macherey & Nagel, Germany) or the Blue Matrix PCR/DNA clean up DNA Purification kit (EurX, Poland), respectively. If gel purification was necessary, we used the QIAquick Gel Extraction kit (Qiagen). In some cases it was required to PCR-clone some fragments for better sequencing results. We then used the pGEM-T Easy Vector system (Promega) and selected positive clones by blue-white screening. Plasmid purification was performed with the Quantum Prep Plasmid mini kit (BioRad), according to the manufacturer's protocol.

**Table 2:** Primer pairs and annealing temperatures successfully used for primer walking.

*Primer Pair*	*Primer Sequence *(3'-5')	*Annealing Temperature *(°C)
SR 16S-f	CTT ATG TTT TTT TTA GTT TGC GAC CTC	62
SR cob-f	GGG GGA GTG ATT GCT TTG TTG C	64
		
SR cox3-f	CAA CAG GGT TTC ACG GAA TAC ACG	65
SR 16S-r	GAG GTC GCA AAC TAA AAA AAA CAT AAG	62
		
SR cobf-rev	TGT TGG AGA TCT AAC AGA ATA AGC AC	60.1
SR co1f-rev	CTT TCT GAG ATA AAA GTA GGT CCT GG	61.6

Sequences are illustrated from 5' to 3' end.

### Sequencing and data assembly

Initial sequencing of amplified PCR fragments was carried out in the Berlin lab, using a CEQ™8000 capillary sequencer (Beckmann-Coulter, USA) and the CEQ DCTS Quick Start kit (Beckmann-Coulter) according to the standard protocol, except for using half volumes for setup of the sequencing reaction (10 μl). Final sequencing was performed by the professional sequencing service of AGOWA (Berlin, Germany). Following sequencing, BLAST programs on the NCBI server were used to determine rRNA- and protein-encoding genes. ClustalW and the cap-contig program, both integrated in BioEdit version 7.0.5 [57], were used for sequence assembly and comparison. The final sequence was compared with previously published mitogenomic sequences of other taxa recovered from GenBank and OGRe [58]. For comparison and evaluation of gene boundaries we built alignments from genes of several metazoan species (predominantly including Platyhelminthes and other Lophotrochozoa, as well as *Xenoturbella bocki*). In addition, our sequence was compared to the recently published EST data of *S. roscoffensis *from the NCBI trace archive to get an independent confirmation of gene boundaries. The putative secondary structures of all tRNAs were either detected in a combined approach using tRNAscan-SE [59], ARWEN [60] or by extensive inspection of intergenic regions by eye. The complete mt genome sequence of *S. roscoffensis *is deposited at the NCBI database with accession number [GenBank: HM237350]. CREx [42] was used to determine common intervals and breakpoint distances in pair wise comparisons of gene orders. AT and GC skew were calculated according to the following formula: AT skew = (A%-T%)/(A%+T%); GC skew = ( G%-C%)/(G%+C%), as described in [35].

### Phylogenetic analysis

For the phylogenetic analyses we concatenated amino acid alignments from 11 protein-coding genes. We omitted alignments from *atp8 *and *nad4l*, as these are the shortest and least conserved genes from the protein-coding set of animal mt genomes. A total of 60 species was chosen to build the alignments. A detailed overview of the respective taxa and their accession numbers is given in table 3. The alignment of protein-coding genes was done with MAFFT using the FFT-NS-i option [61]. Gblocks ver. 0.91 [62] was used for excluding ambiguously aligned proportions. We used the following settings for the 50 species dataset: minimum number for a conserved position: 26; minimum number for a flank position: 26; maximum number of non-conserved positions: 8; minimum length of a block: 10; allowed gap positions: with half. The reduced alignment has a length of 2095 amino acids, which is 42% of the original alignment (4959 amino acids). The alignment is available from the corresponding author's website http://www.cgae.de. NH-PhyloBayes [63] was used to conduct Bayesian inference with the site-heterogeneous CAT model and the BP option, allowing changes of model settings at "breakpoints" along the tree (empirical optimisation). Four independent MCMC chains were run for each specific alignment. Runs were checked for convergence and stopped when all chains converged to a similar topology. If not, runs were continued up to three weeks on a fast multicore processor unit, allowing for about 10000 sample points per run. Bayesian posterior probabilities (BPP) were calculated from the trees sampled during stationary phase of the different chains.

**Table 3:** Names, taxonomic classifications and GenBank accession numbers of the species used in our phylogenetic analyses (asterisks point to partial genome data; percental coverage in the final alignment is indicated for these taxa).

*Species*	*Systematic position*	*GenBank accession number*
*Symsagittifera roscoffensis*	Acoela	[GenBank: HM237350]
Paratomella rubra*(69%)	Acoela	[GenBank: AY228758]
Nemertoderma westbladii*(35%)	Nemertodermatida	[GenBank: AY228757]
*Urechis caupo*	Echiura	[GenBank: NC_006379]
Myzostoma seymourcollegiorum*(80%)	Myzostomida	[GenBank: EF506562]
*Lumbricus terrestris*	Annelida - Clitellata	[GenBank: NC_001677]
*Platynereis dumerilii*	Annelida - "Polychaeta"	[GenBank: NC_000931]
Microstomum lineare*(49%)	Platyhelminthes - Turbellaria	[GenBank: AY228756]
*Trichobillharzia regenti*	Platyhelminthes - Trematoda	[GenBank: NC_009680]
*Fasciola hepatica*	Platyhelminthes - Trematoda	[GenBank: NC_002546]
*Microcotyle sebastis*	Platyhelminthes - Monogenea	[GenBank: NC_009055]
*Gyrodactylus salaris*	Platyhelminthes - Monogenea	[GenBank: NC_008815]
*Diphyllobothrium latum*	Platyhelminthes - Cestoda	[GenBank: NC_008945]
*Hymenolepis diminuta*	Platyhelminthes - Cestoda	[GenBank: NC_002767]
*Trichinella spiralis*	Nematoda	[GenBank: NC_002681]
*Xiphinema americanum*	Nematoda	[GenBank: NC_005928]
*Caenorhabditis elegans*	Nematoda	[GenBank: NC_001328]
*Brachionus plicatilis *(part 1)	Rotifera	[GenBank: NC_010472]
*Brachionus plicatilis *(part 2)	Rotifera	[GenBank:NC_010484]
*Leptorhynchoides thecatus*	Acanthocephala	[GenBank: NC_006892]
*Epiperipatus biolleyi*	Onychophora	[GenBank: NC_009082]
*Limulus polyphemus*	Chelicerata - Xiphosura	[GenBank: NC_003057]
*Lithobius forficatus*	Myriapoda - Chilopoda	[GenBank: NC_002629]
*Drosophila yakuba*	Hexapoda - Pterygota	[GenBank: NC_001322]
*Triops cancriformis*	Crustacea - Phyllopoda	[GenBank: NC_004465]
*Squilla mantis*	Crustacea - Malacostraca	[GenBank: NC_006081]
*Priapulus caudatus*	Priapulida	[GenBank: NC_008557]
*Lineus viridis*	Nemertea	[GenBank: NC_012889]
Phoronis psammophila*(98%)	Phoronida	[GenBank: AY368231]
*Terebratulina retusa*	Brachiopoda	[GenBank: NC_000941]
*Laqueus rubellus*	Brachiopoda	[GenBank: NC_002322]
*Katharina tunicate*	Mollusca - Polyplacophora	[GenBank: NC_001636]
*Haliotis rubra*	Mollusca - Gastropoda	[GenBank: NC_005940]
*Octopus ocellatus*	Mollusca - Cephalopoda	[GenBank: NC_007896]
*Loxosomella aloxiata*	Entoprocta	[GenBank: NC_010432]
*Flustrellidra hispida*	Bryozoa/Ectoprocta	[GenBank: NC_008192]
*Bugula neritina*	Bryozoa/Entoprocta	[GenBank: NC_010197]
*Paraspadella gotoi*	Chaetognatha	[GenBank: NC_006083]
*Spadella cephaloptera*	Chaetognatha	[GenBank: NC_006386]
*Balanoglossus carnosus*	Hemichordata	[GenBank: NC_001887]
*Branchiostoma floridae*	Chordata - Cephalochordata	[GenBank: NC_000834]
*Myxine glutinosa*	Chordata - Craniata	[GenBank: NC_002639]
*Florometra serratissima*	Echinodermata - Crinoidea	[GenBank: NC_001878]
*Asterias amurensis*	Echinodermata - Asteroidea	[GenBank: NC_006665]
*Cucumaria miniata*	Echinodermata - Holothuroidea	[GenBank: NC_005929]
*Xenoturbella bocki*	Xenoturbellida	[GenBank: NC_008556]
*Oscarella carmela*	Porifera - Demospongia	[GenBank: NC_009090]
*Geodia neptuni*	Porifera - Demospongia	[GenBank: NC_006990]
*Acropora tenuis*	Cnidaria - Anthozoa	[GenBank: NC_003522]
*Aurelia aurita*	Cnidaria - Scyphozoa	[GenBank: NC_008446]
*Trichoplax adhaerens*	Placozoa	[GenBank: NC_008151]

## Abbreviations

*atp 6/8*: ATPase subunit 6/8 genes; *cob*: cytochrome b gene; *cox 1-3*:cytochrome oxidase subunit I-III genes; mtDNA: mitochondrial DNA; *nad1-6 *and *nad4L*: NADH dehydrogenase subunit 1-6 and 4L genes; *rrnS/rrnL*: small/large rRNA subunit genes; rRNA: ribosomal RNA; tRNA: transfer RNA; *trnX*: tRNA gene X ('X' replaces the one-letter amino acid code of the respective tRNA); nt: nucleotides; PCR: polymerase chain reaction

## Authors' contributions

AM did most of the laboratory experiments. XB reared animals and provided helpful EST data. All authors analysed parts of the data and did phylogenetic analyses. All authors discussed results. AM, LP and DTJL wrote substantial parts of the manuscript. All authors read and approved the final manuscript.

## Supplementary Material

Additional file 1 **Supplemental Figs. S1-3**. Gene order comparison with CREX (number of common intervals, breakpoint distances); phylogenetic analysis under exclusion of Acoela and Nemertodermatida; phylogenetic analysis under exclusion of *Xenoturbella*. Click here for file
