# Supplementary material for: The phylogenetic position of Acoela as revealed by the complete mitochondrial genome of Symsagittifera roscoffensis
Source: BMC Evol Biol. 2010 Oct 13;10:309. doi: 10.1186/1471-2148-10-309 (PMC2973942; doi:10.1186/1471-2148-10-309)
Supplement: Additional file 1 — Supplemental Figs. S1-3. Gene order comparison with CREX (number of common intervals, breakpoint distances); phylogenetic analysis under exclusion of Acoela and Nemertodermatida; phylogenetic analysis under exclusion of Xenoturbella. [file 1471-2148-10-309-S1.PDF]

Common interv.	D	X	T	P	A	A	S	U	N	T	P	K	F	B	A	F	S	S	P	S
Deuterostomia	204	134	44	68	104	26	32	16	56	50	40	46	0	14	18	10	8	16	14	14
Xenoturbella	134	204	34	56	68	20	28	14	38	50	40	46	6	20	30	8	10	18	14	14
Trichinella	44	34	204	54	38	20	22	14	20	26	28	24	4	12	10	10	8	12	24	12
Priapulus	68	56	54	204	108	28	34	28	54	78	86	92	0	14	14	6	8	14	12	10
Arthropoda	104	68	38	108	204	26	20	14	62	92	72	78	0	14	16	6	6	12	12	10
Annelida	26	20	20	28	26	204	52	46	38	36	34	44	6	10	12	18	8	6	32	20
Sipunculus	32	28	22	34	20	52	204	26	20	20	22	24	0	6	14	6	14	8	6	4
Urechis	16	14	14	28	14	46	26	204	14	14	22	34	0	10	4	12	8	4	10	12
Nemertea	56	38	20	54	62	38	20	14	204	106	52	70	2	26	18	16	10	8	16	14
Terebratulina	50	50	26	78	92	36	20	14	106	204	90	108	8	24	14	16	18	4	20	16
Phoronis	40	40	28	86	72	34	22	22	52	90	204	106	6	34	16	10	10	6	24	22
Katharina	46	46	24	92	78	44	24	34	70	108	106	204	6	32	14	10	10	2	24	22
Flustrellidra	0	6	4	0	0	6	0	0	2	8	6	6	204	16	2	8	8	6	4	18
Bugula	14	20	12	14	14	10	6	10	26	24	34	32	16	204	26	10	10	2	16	14
Acanthocephala	18	30	10	14	16	12	14	4	18	14	16	14	2	26	176	8	12	8	14	16
Fasciola	10	8	10	6	6	18	6	12	16	16	10	10	8	10	8	176	60	2	10	6
Schistosoma man.	8	10	8	8	6	8	14	8	10	18	10	10	8	10	12	60	176	0	8	4
Symsagittifera	16	18	12	14	12	6	8	4	8	4	6	2	6	2	8	2	0	176	10	4
Paraspadella	14	14	24	12	12	32	6	10	16	20	24	24	4	16	14	10	8	10	150	46
Spadella	14	14	12	10	10	20	4	12	14	16	22	22	18	14	16	6	4	4	46	150

BP distance	D	X	T	P	A	A	S	U	N	T	P	K	F	B	A	F	S	S	P	S
Deuterostomia	0	3	8	5	5	9	8	11	6	6	8	7	15	11	10	13	13	12	12	13
Xenoturbella	3	0	8	6	6	10	8	11	7	7	9	8	14	11	9	13	13	12	12	13
Trichinella	8	8	0	8	8	11	10	11	11	10	10	10	14	12	12	12	13	13	10	11
Priapulus	5	6	8	0	2	8	8	9	6	5	7	6	15	11	11	13	13	13	11	12
Arthropoda	5	6	8	2	0	8	9	10	6	5	7	6	15	11	11	13	13	13	11	12
Annelida	9	10	11	8	8	0	6	6	8	7	8	8	13	12	11	12	13	12	9	9
Sipunculus	8	8	10	8	9	6	0	8	10	9	9	9	15	13	10	13	13	11	11	12
Urechis	11	11	11	9	10	6	8	0	11	10	10	10	15	13	13	11	13	13	10	10
Nemertea	6	7	11	6	6	8	10	11	0	3	7	6	14	9	11	12	12	11	11	12
Terebratulina	6	7	10	5	5	7	9	10	3	0	5	4	13	9	11	11	11	12	10	11
Phoronis	8	9	10	7	7	8	9	10	7	5	0	3	14	10	11	12	12	13	10	11
Katharina	7	8	10	6	6	8	9	10	6	4	3	0	14	9	11	12	12	13	10	11
Flustrellidra	15	14	14	15	15	13	15	15	14	13	14	14	0	12	14	14	14	14	12	12
Bugula	11	11	12	11	11	12	13	13	9	9	10	9	12	0	10	12	12	13	11	11
Acanthocephala	10	9	12	11	11	11	10	13	11	11	11	11	14	10	0	11	10	11	13	13
Fasciola	13	13	12	13	13	12	13	11	12	11	12	12	14	12	11	0	5	14	12	12
Schistosoma-mansoni	13	13	13	13	13	13	13	13	12	11	12	12	14	12	10	5	0	14	13	13
Symsagittifera	12	12	13	13	13	12	11	13	11	12	13	13	14	13	11	14	14	0	10	12
Paraspadella	12	12	10	11	11	9	11	10	11	10	10	10	12	11	13	12	13	10	0	5
Spadella	13	13	11	12	12	9	12	10	12	11	11	11	12	11	13	12	13	12	5	0

Supplementary material, Figure S1. Mitochondrial gene order (excluding tRNAs). Pairwise comparison of common intervals (above), defined as number of shared gene blocks, independent from gene order inside a block [42] and breakpoint distance (below) among Bilateria.

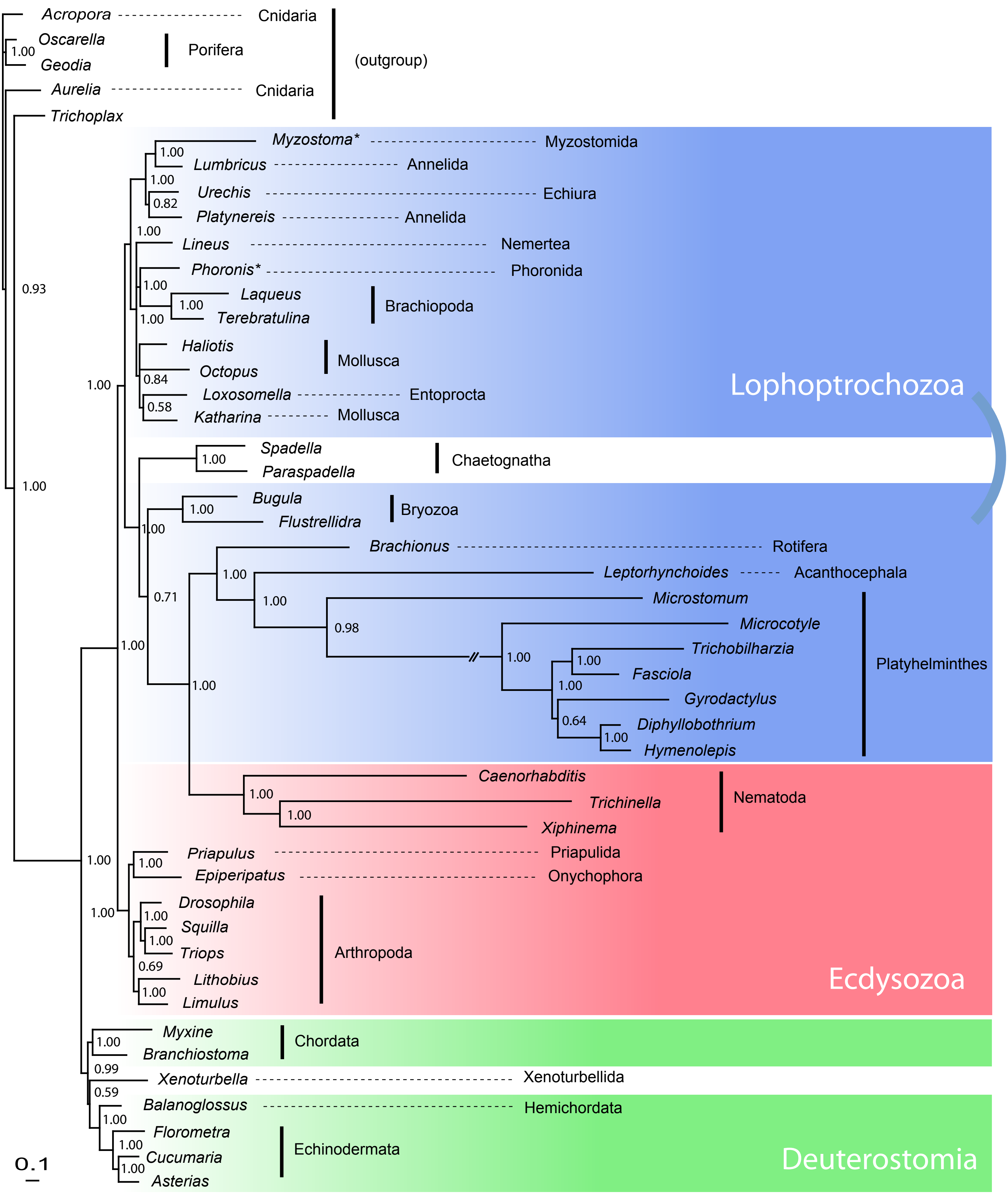

Supplementary material, Figure S2. Tree topology resulting from phylogenetic analysis. NH-PhyloBayes, CAT -BP model. The alignment differs from that presented in the main body of the manuscript by the exclusion of Acoela and Nemertodermatida.

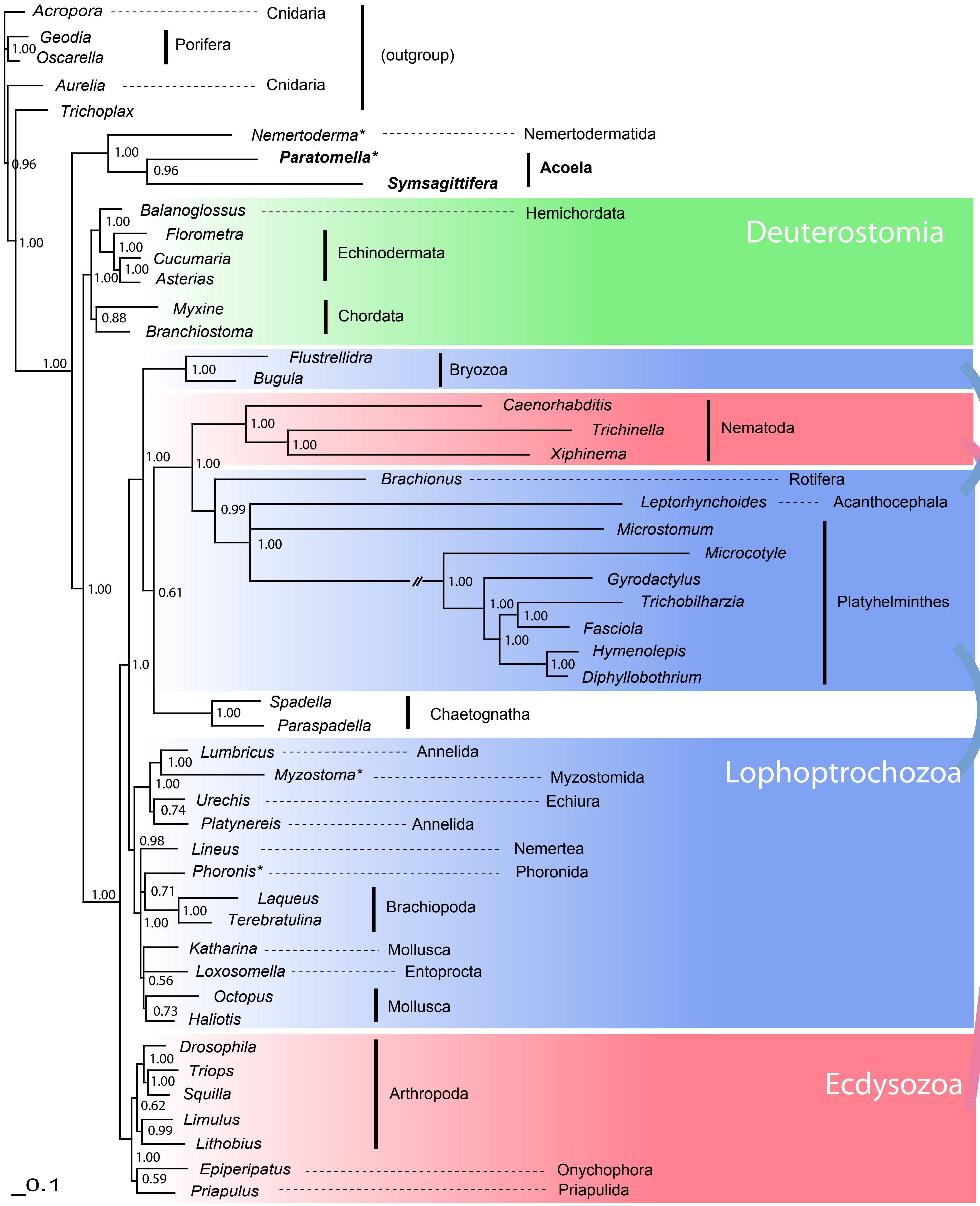

Supplementary material, Figure S3. Tree topology resulting from phylogenetic analysis. NH-PhyloBayes, CAT -BP model. The alignment differs from that presented in the main body of the manuscript by the exclusion of *Xenoturbella bocki*.
